# A case of eruptive keratoacanthomas and lichen planus secondary to nivolumab treatment for stage IV melanoma

**DOI:** 10.1016/j.jdcr.2024.10.037

**Published:** 2024-12-26

**Authors:** Daniel S. Alicea, Bijal Amin, Beth N. McLellan

**Affiliations:** a Division of Dermatology, Department of Medicine, Albert Einstein College of Medicine, Montefiore Medical Center, Bronx, New York; b Department of Pathology, Albert Einstein College of Medicine, Montefiore Medical Center, Bronx, New York

**Keywords:** immune-checkpoint inhibitors, keratoacanthoma, lichen planus, melanoma, nivolumab, squamous cell carcinoma

## Introduction

Immune-checkpoint inhibitors (ICIs) are a form of immunotherapy that has transformed oncologic management, yet is often linked to the occurrence of various immune related adverse events including cutaneous toxicities. While the most reported skin findings include pruritus, eczema, lichenoid drug reactions, and vitiligo, other skin toxicities, such as lichen planus and keratoacanthoma, are rare with limited documented cases and treatment recommendations limited to case reports and case series. There is no consensus on optimal treatment, especially in the setting of cases that require alternative strategies that target multiple skin adverse events. Here, we present the unique combination of both lichen planus (LP) and keratoacanthoma (KAs) in a patient who received nivolumab for metastatic melanoma to the lung which responded excellently to dupilumab and intralesional 5-fluorouracil.

## Case report

**Fig 1:**
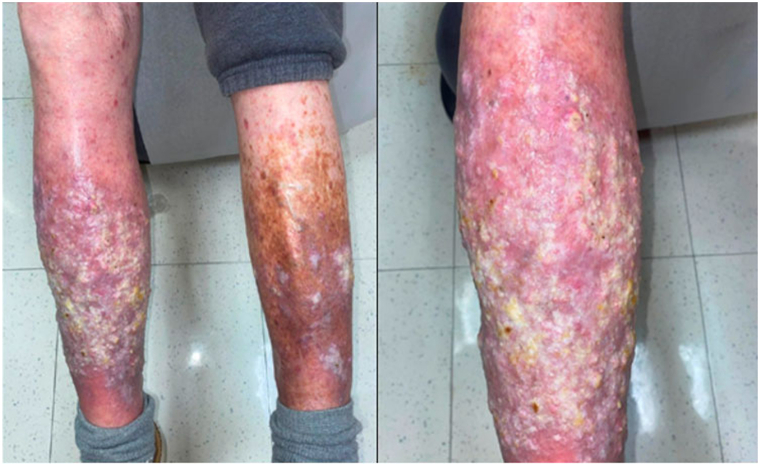
*Pink* lichenoid plaques with thick *yellow* adherent keratotic nodules and background erythema on the bilateral legs consistent with hypertrophic lichen planus and possible keratoacanthoma or squamous cell carcinoma.

A 77-year-old male with a past medical history of multiple squamous cell carcinomas (SCCs) on the head, neck, bilateral upper and lower extremities, basal cell carcinoma on the head and bilateral lower extremities, metastatic melanoma to the lung, and severe sunburns as a child, on treatment with single-agent nivolumab 240 mg intravenous infusions every 4 weeks for 8 months, presented to dermatology for a rash on the legs. The patient had been previously prescribed a class 1 topical corticosteroid with suboptimal improvement in pruritus and appearance of the rash. Nivolumab, an ICI, was held due to the rash. The patient denied systemic symptoms. Examination revealed pink-violaceous plaques with crust and keratotic scale on the bilateral legs with moderate to severe photodamaged skin of the head, neck, upper, and lower bilateral extremities. Differential diagnoses included hypertrophic LP and possible KA or SCC (Fig 1).

**Fig 2:**
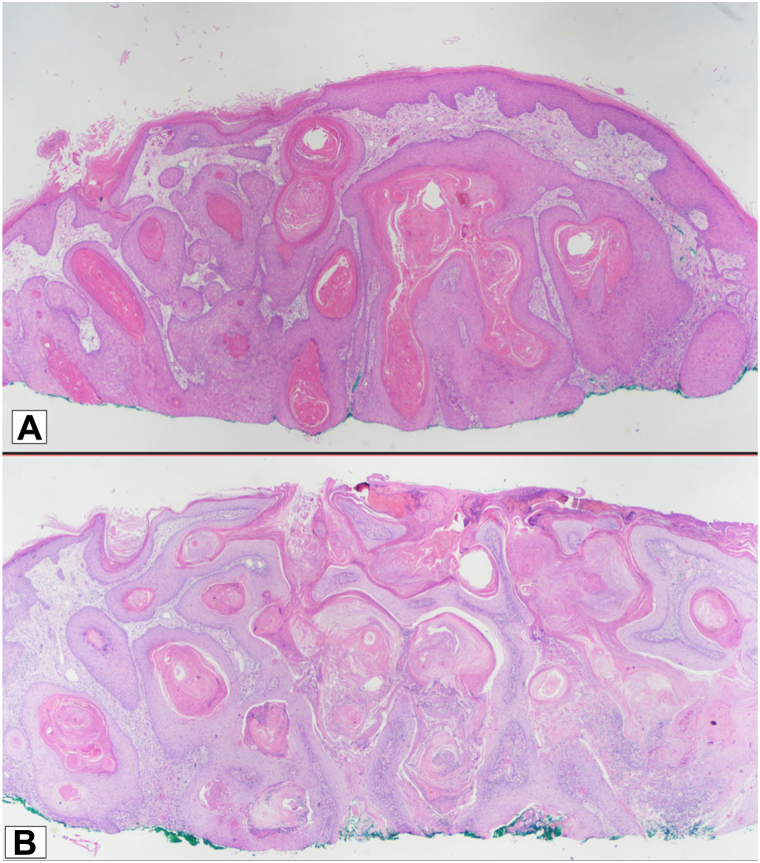
**A** and **B**, Crateriform squamous proliferation with abundant keratin and associated lymphocytic inflammation consistent with a keratoacanthoma, a well-differentiated squamous cell carcinoma, or hypertrophic lichen planus at the specimen's periphery (H&E, 2×).

Shave biopsies from the right leg 2 months prior demonstrated the surface of an atypical squamous epithelial proliferation and atypical endophytic squamous proliferation consistent with either a KA, a well-differentiated SCC, or hypertrophic LP at the specimen's periphery (Fig 2, *A* and *B*). Given the concerns for KAs with a background of what appeared clinically to be LP, the patient was started on acitretin 10 mg daily. The patient had improved after 1 month on acitretin with less scale and erythema; however, when the dose was escalated to 25 mg daily, the patient chose to discontinue the medication completely due to perceived side effects of body aches and headaches.

Due to lack of alternative treatments that could manage both the LP and KAs, we elected to start dupilumab to treat the ICI-induced LP first and then address the KAs after background inflammation had improved. Dupilumab was initiated with a loading dose of 600 mg and then 300 mg every other week.

**Fig 3:**
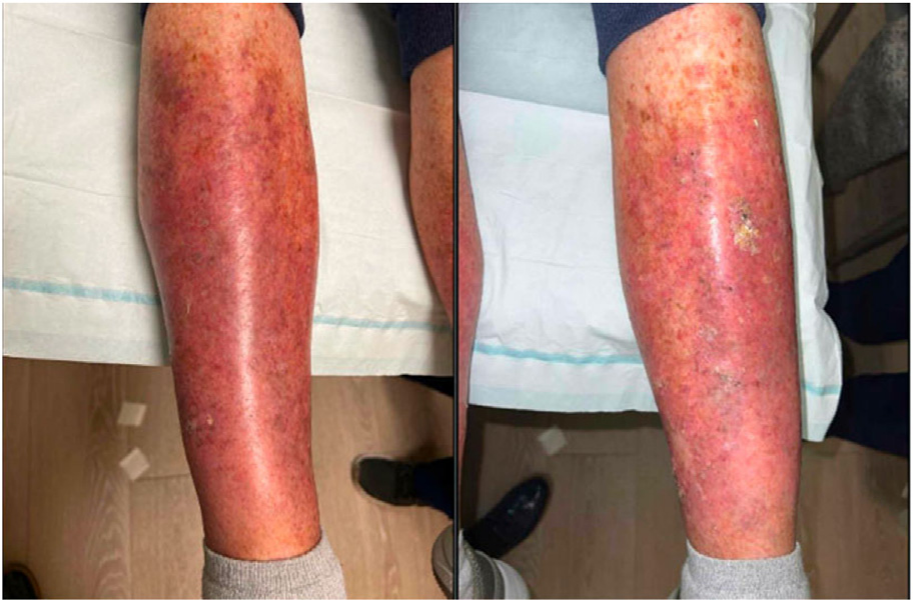
Marked improvement in keratoacanthoma appearance after 5-fluorouracil treatment with residual erythema and a few thick *yellow* adherent keratotic nodules on the bilateral legs.

There was an improvement in pruritus 1 week following initiation of dupilumab, and there was continued subsequent improvement of erythema and scale on the legs although the discrete keratotic nodules persisted. At that point, we initiated intralesional 5-fluorouracil 25 mg to 1 KA on the right leg as a test dose. Three weeks later, the treated KA showed improvement with softening and regression of the lesion. This prompted treatment of the remaining KAs with 3 total injections of intralesional 5-fluorouracil 50 mg/mL which all regressed (Fig 3). The treating oncologist elected not to resume nivolumab given combination of these adverse events and the continued excellent response seen on magnetic resonance imaging of the brain and computerized tomography scan of the chest, abdomen, and pelvis.

## Discussion and conclusion

Cutaneous immune related adverse events (iRAEs) are common among patients receiving ICI therapy, with the most reported skin findings being pruritus, eczema, lichenoid drug reactions, and vitiligo.^1^ Anti-programmed cell death protein 1 (PD-1) and programmed death-ligand 1 (PD-L1) agents, like nivolumab, inhibit a transmembrane protein known as PD-1 which is expressed on T-cells, B-cells, and natural killer-cells. These ICIs function by counteracting tumor cell inhibition of T-cells, thus producing an antitumor response. However, blockage of PD-1 and PD-L1 leads to T cell overactivation, resulting in iRAEs.^2^ In fact, despite the favorable safety profile associated with monoclonal antibodies targeting PD-1 and PD-L1, dermatological toxicities affect more than 30% of treated patients.^3^ According to the Common Terminology Criteria for Adverse Events, most cutaneous toxicities from ICIs are primarily grade 1, defined as asymptomatic to mild, or 2, defined as moderate in severity.^2^ Because most cutaneous iRAEs are low grade, ICI treatment rarely leads to a dose reduction or discontinuation. The combination of LP and eruptive KAs secondary to PD-1 inhibitors have been previously described in the literature^4^, ^5^, ^6^; however, documented cases are limited, and treatment recommendations are primarily based on case reports and case series. KAs remain as a rare cutaneous adverse effect of ICI therapy, with 22 reported cases in the literature from PD-1/PD-L1 inhibitor usage.^5^ KAs have been described as low-risk histologic variants of SCCs and develop in favorable microenvironments of predisposed individuals, such as those with sun exposure or ultraviolet radiation.^7^ Thus, it has been postulated that ICI therapy in heavily ultraviolet-exposed patients are at an increased risk of developing squamoproliferative cutaneous lesions. The histopathologic differentiation of hypertrophic LP-associated squamous atypia from malignant KA/SCC can be challenging and often requires a thorough clinical history, along with close collaboration and communication between dermatologists and dermatopathologists. In our case, the dermatologist's clinical suspicion, together with subtle findings identified upon re-review of the biopsies, provided sufficient grounds to initiate appropriate treatment(s), and the patient's favorable clinical response(s) further supported the respective diagnoses. We report the unusual combination of PD-1 induced LP and KA in a patient with metastatic melanoma to the lung who showed an excellent response to dupilumab and intralesional 5-fluorouracil.

## Conflicts of interest

None disclosed.
